# Polar Climate Change as Manifest in Atmospheric Circulation

**DOI:** 10.1007/s40641-018-0111-4

**Published:** 2018-08-02

**Authors:** J. A. Screen, T. J. Bracegirdle, I. Simmonds

**Affiliations:** 1 University of Exeter, 920 Laver Building, North Park Road, Exeter, Devon EX4 4QE UK; 2 British Antarctic Survey, Cambridge, UK; 3 The University of Melbourne, Melbourne, Australia

**Keywords:** Arctic, Antarctic, Climate change, Stratospheric polar vortex, Annular modes, Cyclones

## Abstract

**Purpose of Review:**

Dynamic manifestations of climate change, i.e. those related to circulation, are less well understood than are thermodynamic, or temperature-related aspects. However, this knowledge gap is narrowing. We review recent progress in understanding the causes of observed changes in polar tropospheric and stratospheric circulation, and in interpreting climate model projections of their future changes.

**Recent Findings:**

Trends in the annular modes reflect the influences of multiple drivers. In the Northern Hemisphere, there appears to be a “tug-of-war” between the opposing effects of Arctic near-surface warming and tropical upper tropospheric warming, two predominant features of the atmospheric response to increasing greenhouse gases. Future trends in the Southern Hemisphere largely depend on the competing effects of stratospheric ozone recovery and increasing greenhouse gases.

**Summary:**

Human influence on the Antarctic circulation is detectable in the strengthening of the stratospheric polar vortex and the poleward shift of the tropospheric westerly winds. Observed Arctic circulation changes cannot be confidently separated from internal atmospheric variability.

## Introduction

The planet is unequivocally warming due to human influence [1]. Whilst the basic physics of global warming is clear, the response of the atmospheric circulation at the regional scale is less well known [2, 3]. This poses a challenge to the attribution of past climate change to human influence and is a large source of uncertainty in climate projections. As Shepherd (2014) puts it: “nearly everything we have any confidence in when it comes to climate change is related to global patterns of surface temperature, which are primarily controlled by thermodynamics. In contrast, we have much less confidence in atmospheric circulation aspects of climate change, which are primarily controlled by dynamics and exert a strong control on regional climate” [2]. This is especially true for the high latitudes, where internal variability of the atmospheric circulation is a major source of uncertainty in projected climate change [4].

This review forms part of a Topical Collection on Climate Change and Atmospheric Circulation. Here, we focus on the polar regions, but also consider relevant aspects of the mid-latitude atmospheric circulation that are intimately connected to changes in the polar regions. Other papers in this Collection cover the tropical convergence zones [5], monsoons [6], subtropical highs [7] and blocking [8]. Our review is not intended to cover all aspects of the polar circulation but, instead, focuses on selected areas of research where significant scientific advances have been made in the past 5 years. In the following sections, we report on progress in understanding the past and projected future climate change response of the following atmospheric circulation phenomena: the northern and southern stratospheric polar vortices, Arctic and sub-Antarctic cyclones, Arctic moist intrusions, and the Amundsen Sea Low. We also consider the dominant modes of atmospheric circulation variability in the extratropics of each hemisphere, namely the Northern Annular Mode and Southern Annular Mode. Rather than describing each of these circulation features now, we provide some background within the relevant sections. We deal first with the Arctic, then the Antarctic, and lastly, reflect on the differences and similarities between circulation changes in the two polar regions.

## The Arctic

### Northern Annular Mode

The Northern Annular Mode (NAM), also known as the Arctic Oscillation, is the dominant mode of inter-annual variability in the extratropical Northern Hemisphere [9]. The positive phase of the NAM is associated with anomalously low pressure over the Arctic and high pressure over mid-latitudes, a pattern that strengthens the zonally averaged westerly winds. Also, the westerly winds are typically located further poleward during positive NAM. Since changes in the westerly winds, storm tracks and the eddy-driven jet stream are intimately connected, both to each other and to the NAM, we will consider these collectively here.

In the past few years, there has been considerable progress in understanding the role of Arctic sea ice as a driver of variability in the NAM. Interest in links between sea ice and the NAM has been sparked by the repeated coincidence of anomalously negative NAM and reduced sea ice conditions and the dramatic decline in Arctic sea ice. Several studies have noted a strong negative correlation between sea ice and the NAM over recent decades [10–13]. Given that attributing causality is an intractable problem with observations alone, many studies have reported on model experiments with perturbed sea ice to isolate the atmospheric circulation response to sea ice. These modelling studies support a causal link between projected sea ice loss and the negative NAM [14••, 15–18, 19•, 20••]. A recent comparison of the atmospheric circulation response to projected Arctic sea ice loss in six coupled climate model experiments found a weakening and equatorward shift of the midlatitude westerly wind belt in each case [21], implying a common negative NAM response. However, the NAM response to observed sea ice loss over the past 30–40 years is less clear, being model dependent and often obscured by internal variability [22]. More detailed reviews on the possible links between Arctic sea ice loss and the Northern Hemisphere atmospheric circulation can be found elsewhere [23–27].

Sea ice loss can force a change in the NAM via a tropospheric pathway or through interaction with the stratosphere [28]. The tropospheric pathway is associated with the adjustment of transient eddies to the change in meridional temperature gradient and baroclinic instability. The stratospheric pathway involves enhanced upward planetary-scale wave propagation and the weakening of the stratospheric polar vortex [29, 30], which then modifies the NAM. Idealised atmospheric general circulation model experiments, using configurations with and without an interactive stratosphere, suggest that when the stratosphere is inactive, the tropospheric jet still shifts equatorward in response to Arctic warming, but by approximately half the magnitude compared to that of an active stratosphere [31]. The stratospheric pathway appears sensitive to the location of sea ice loss whereas the tropospheric pathway appears less so [28, 32].

**Fig. 1:**
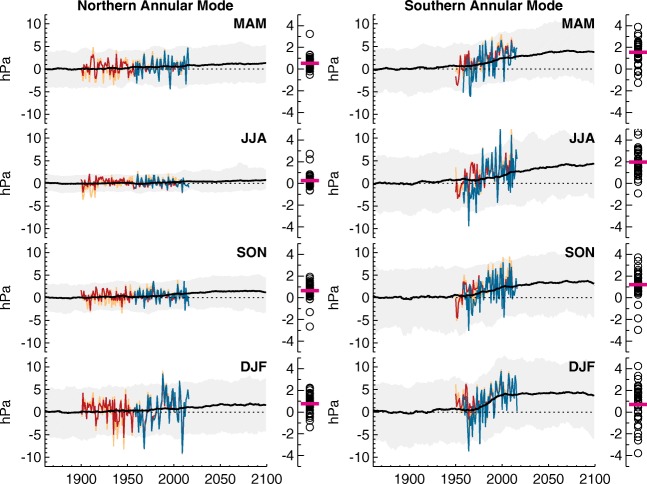
Indices of the Northern Annular Mode (left) and Southern Annular Mode (right) from observations and models. The black line shows, for each season (top to bottom), the CMIP5 multi-model mean of historical and RCP4.5 simulations. The grey band shows the 5–95% confidence range based on the individual model simulations. The coloured lines show observational indices derived from HadSLP2 (red), NOAA-CIRES Twentieth Century Reanalysis (orange) and Japanese 55-year Reanalysis (blue). Simulated anomalies are shown relative to the 1861–1900 baseline, and observations are centred on the multi-model mean over the period for which they are shown. The symbols to the right of each line graph show changes between 1980–2029 and 2050–2099, for each individual model (black circles) and the multi-model mean (red line). Adapted from Gillett and Fyfe (2013), where further details on these data and methods can be found [34]

Arctic sea ice loss is only one component of greenhouse gas (GHG)-induced climate change. A paradigm that has gained traction in recent years is that the climate response to sea ice loss may partly counteract other aspects of the response to increased GHG. Since the dominant characteristic of projected GHG-induced climate change, in the absence of sea ice loss, is pronounced warming of the tropical upper troposphere, this has been conceptualised as a “tug-of-war” between the Arctic and tropics. Whilst Arctic sea ice loss is thought to promote the negative NAM, projected tropical upper tropospheric warming has the opposite effect, favouring the positive NAM [33]. The winter atmospheric responses to Arctic sea ice loss and to GHG increases appear to be distinct, separable and, to good approximation, linearly additive [19•]. The weak projected climate change response of the NAM (Fig. 1) may reflect the partial compensation between the distinct—and opposing—responses to Arctic sea ice loss and GHG increases [14••, 15, 17, 18, 19•, 35•]. This tug-of-war paradigm has been used to explain across-model spread in winter NAM projections [36]. More specifically, models that simulate larger Arctic warming are also those that simulate larger equatorward shifts of the storm tracks [37, 38] and jet stream [39, 40].

### Northern Stratospheric Polar Vortex

The dominant circulation feature of the Arctic stratosphere in winter is a strong circumpolar westerly jet, referred to as the stratospheric polar vortex [41]. Whilst the vortex is omnipresent in winter and centred around the pole, it exhibits large variability in strength and location on intra-seasonal to multi-decadal timescales. It is well known that this stratospheric variability has a significant influence on tropospheric circulation, and weather and climate at the Earth’s surface [41]. Sudden stratospheric warming (SSW) events in particular, when the stratospheric polar vortex is abruptly weakened and disrupted, are often a precursor to the negative phase of the NAM and cold-air outbreaks. Several recent studies have reported a weakening of the polar vortex over the last three or four decades [42–44]. Kretschmer et al. (2018) identified occurrences of weak and strong polar vortices using a hierarchical clustering approach [44]. They found a significant increase in the frequency of the weak vortex events, from an average frequency of 3 days per winter in the period 1979–1996 to 7 days per winter in the period 1998–2015. In contrast, the occurrence of strong vortex events halved from 12 to 6 days over the same years. SSW events have also been notably more frequent in the more recent period [43]. This weakening of the polar vortex has been cited as a cause of increased cold extremes and sustained boreal winter cooling over midlatitude Eurasia [42, 44]. An apparent shift of the polar vortex towards the Eurasian continent and away from North America may have also contributed to cooling over Eurasia [45].

The causes of observed polar vortex weakening are not fully understood. One hypothesised cause is the loss of Arctic sea ice [28–30, 45]. Kim et al. (2014) conducted atmospheric model simulations with reduced sea ice in the Barents and Kara Seas and found an increase in vertical wave activity propagation into the stratosphere [28]. Enhanced wave breaking in the stratosphere caused a deceleration of the stratospheric westerly winds and hence, weakened the polar vortex. Although a general weakening of the polar vortex has been noted in many model experiments with perturbed Arctic sea ice [28–30], the attribution of the observed change to sea ice loss is complicated by at least three factors. The first is divergence between different models [21]. Second, there is an apparent sensitivity to the geographical location of ice loss. In model experiments, sea ice loss in the Atlantic sector appears to cause a weaker vortex, whereas a stronger vortex is found in response to sea ice loss in the Pacific sector [31, 32].

The third complicating factor is internal variability. Two recent studies argue that the observed polar vortex weakening is a manifestation of internal variability and not a forced response to Arctic sea ice loss, or to climate change more broadly. Seviour (2017) examined large ensembles of coupled climate model simulations and concluded that the forced response of the vortex was small relative to internal variability, and that vortex trends of similar magnitude to those observed can be generated purely by internal climate variability [43]. The latter conclusion was also reached by Garfinkel et al. (2017), who found no consistent change in the strength of the polar vortex in model simulations forced with observed GHG concentrations, sea surface temperatures and sea ice [42]. However, unforced internal variability was sufficiently strong that some individual ensemble members reproduced the observed polar vortex weakening.

Future projections of the stratospheric polar vortex are highly uncertain. For example, under global warming scenarios, some modelling studies find an increased frequency of SSW events but others do not [46, 47]. A recent reassessment of projected changes in SSW events, based on 12 models participating in the Chemistry Climate Model Initiative, found no evidence of future changes in the frequency or duration of SSW events [48].

### Moist Intrusions

An aspect of Arctic meteorology that has garnered considerable scientific interest in recent years is intrusions of warm and moist air. Intrusions occur predominantly over the ocean basins and typically when a large-scale blocking pattern to the east of each basin deflects midlatitude cyclones and their associated moisture into the Arctic [49]. Inter-annual variability of intrusions is strongly related with Arctic surface temperature, through their effect on the downward longwave radiation [50, 51]. In extreme cases, the passage of strong cyclones into the Arctic and their associated moist intrusions can lead to above-freezing temperatures in the central Arctic in the midst of winter [52, 53]. In recent years, a run of such Arctic midwinter warming events has focussed attention of whether intrusions are becoming more common [53, 54]. Woods and Caballero (2016) found an increase in the frequency of moist intrusions, which can explain roughly 45% of the surface warming trend and 30% of the sea ice loss in the Barents Sea during the past two decades [50]. Park et al. (2015) concluded that nearly half the sea ice decline in the Atlantic sector could be explained by increased downward longwave radiation, related to increased poleward transport of heat and moisture [51].

Moisture transport into the Arctic is projected to increase in a warming climate, as humidity increases more rapidly at lower latitudes per Kelvin temperature rise, leading to a larger poleward moisture gradient. Skific and Francis (2013) diagnosed that 75–80% of the total projected annual change in moisture transport across 70°N, between the late 20th and 21st centuries, is thermodynamically driven, being due to change in the meridional gradient of specific humidity [55]. Although smaller, the dynamic term was also positive and related to an increase in low-pressure systems over the central Arctic that transport substantial moisture into the Arctic.

### Arctic Cyclones

A ubiquitous aspect of the polar and sub-polar regions of both hemispheres is the presence of many synoptic systems. These play a central role in transporting heat and moisture from the midlatitudes into the polar regions. In recent times, the analysis of cyclonic activity has attracted increased attention. Part of the reason for this is that reduced sea ice is allowing increased human activity in the Arctic, for example shipping, and for which storminess is an important consideration. An issue that arises in this context is that there is no clear objective definition of what comprises a cyclone or its intensity. The fact that there are numerous state-of-the-art automated cyclone identification algorithms available reflects the existence of this ambiguity. Detection algorithms based on different physical assumptions and approaches have been shown to produce broadly consistent findings but with some deviations [56]. A related matter is that a cyclone investigation can be undertaken with a choice of reanalysis data set. The basic fields (e.g. mean sea level pressure) are, in the main, quite coherent across the sets, but they can show sizeable deviations in the vicinity of cyclones and fronts. Hence, when discussing diagnosed cyclone behaviour, it is important to appreciate what identification scheme was applied, and what reanalysis data set was used. These issues may be of particular relevance when undertaking cyclone analysis in the Arctic region. Simmonds and Rudeva (2014) found that 10 state-of-the-art cyclone identification schemes, applied to intense systems in the ERA-Interim reanalysis, were in substantial agreement as to the location of the centre of these storms, while showing considerable differences between the central pressures diagnosed by the algorithms of typically 5–10 hPa [57].

Rudeva and Simmonds (2015) analysed cyclone behaviour in 34 years of data and found significant increases in winter cyclone frequency to the north of the Canadian Arctic Archipelago and to the north of Greenland [58]. In summer, they revealed a quite different pattern of change, with negative trends north of the Beaufort Sea and positive trends centred over the Laptev Sea. Similar trend results were achieved by Zahn et al. (2018) who used a somewhat shorter period and four reanalyses [59]. They identified increasing winter cyclone frequency over most of the central Arctic Ocean and towards the Pacific, and decreasing winter cyclone frequency in the Barents Sea and north of the Russian coast. These cyclone frequency changes are consistent with observed winter changes in the frequency and longevity of high-latitude blocking [60]. The contrasting regional trends were broadly consistent across the four reanalyses. They showed, however, that the magnitudes of trends differed in strength between reanalyses. When summed over the entire Arctic basin, the number of Arctic cyclones exhibits no significant linear trend in any season [61, 62]. Given the opposing regional trends mentioned above, this finding is perhaps not surprising.

Arctic cyclone characteristics are correlated with key large-scale atmospheric indices, such as El Niño Southern Oscillation (ENSO) and NAM; however, these associations vary with season and none alone sufficiently explains cyclone variability or trends [58, 61]. The role that decreasing Arctic ice may be playing in these cyclone trends is a matter of considerable debate. Interaction between variability in Arctic sea ice and cyclones are complex, and apparent ice-cyclone associations appear dependent on data set resolution, and the identification method used [63•]. Open water areas in the Arctic have led to increased transfer of heat and moisture from the ocean to the atmosphere, warming and moistening the Arctic atmosphere and increasing the *potential* for cyclogenesis [64]. However, the effect of atmospheric moisture increases on cyclone behaviour is unclear and appears sensitive to the spatial resolution of the reanalysis dataset and/or how well current models represent moist processes [64–69]. What is becoming clearer is that investigations undertaken with higher resolution data sets reveal more cyclonic structures [70•, 71]. For example, Tilinina et al. (2014) conducted a cyclone investigation using the high-resolution Arctic System Reanalysis and identified a considerably higher number of cyclones over the Arctic [70•]. They found the set allowed for more accurate description of the life cycle of the most intense Arctic cyclones, compared to the global reanalyses. At higher resolution, cyclones had lower central pressures, faster deepening and stronger winds on average. Trends in such intense Arctic cyclones are attracting much interest, and there are suggestions that these features are becoming more frequent [72]. The so-called Great Arctic Cyclone of August 2012 [73] broke the then-record of the lowest central pressure of all Arctic August storms since 1979 and this record still stands.

Turning now to future projections of Arctic cyclones, Day et al. (2018) conducted experiments with the CESM1-CAM5 climate model under a high GHG emission scenario [74••]. They compared Arctic cyclone properties during 2071–2080 with those revealed over 1990–2005 in their historical simulation and found the response to vary with season. In winter, they found a significant reduction in both the number and intensity of storms, and a strong decrease in the frequency with which strong cyclones occur. The cyclone decrease was particularly marked in a band from Greenland to the Norwegian Sea, consistent with observed increases in high-latitude blocking near Greenland [75, 76]. By contrast they identified an increase in cyclone activity in the summer season and a rise in the frequency of strong storms. Their analysis allowed them to posit that this strong seasonality was associated with changes in the high-latitude meridional temperature gradient. In winter, this gradient is weakened as a result of amplified Arctic warming, while it is strengthened in summer because of the enhanced warming of high latitude continents compared to the Arctic Ocean.

A similar analysis, but using a different cyclone identification scheme, was conducted by Crawford and Serreze (2017) but they focussed on the summer season and on changes in the Arctic frontal zone [77]. This zone is a tight band of strong horizontal temperature gradients and hence, baroclinicity, which develops along the Arctic Ocean coastline. They found that the strengthening of the frontal zone in June is accompanied by increases in cyclone activity along some parts of this zone, but little change in cyclone numbers over the Arctic basin.

The complex role of baroclinicity has been further explored by Tamarin-Brodsky and Kaspi (2017) in connection with the poleward shift, from 1980–1999 to 2080–2099, of storm tracks in climate models forced by increased GHG emissions from the Coupled Model Intercomparison Project phase 5 (CMIP5) [78••]. They demonstrated that cyclones are not only formed more poleward in a warmer climate, but they also propagate more poleward until they reach their peak intensity. However, within the Arctic basin, they show that the CMIP5 models exhibit a winter decrease in cyclone numbers.

Regional climate models have also been used to investigate possible future change in Arctic cyclone characteristics. Cyclone changes from 1980–1999 to 2080–2099 were modelled by Akperov et al. (2015) using the HIRHAM model under a midrange GHG emission scenario [79]. They found greater Arctic cyclone numbers in the warm season and fewer in the cool season, though neither of these changes was statistically significant. However, they detected clear changes in intensity and size of cyclones for both seasons.

In summary, observed and projected changes in the Arctic atmospheric circulation are of fairly modest magnitude compared to naturally occurring climate variability. We now turn our attention to the Southern Hemisphere, where the combined effects of GHG increases and stratospheric ozone depletion have led to clearer circulation changes over recent decades.

## The Antarctic

### Southern Annular Mode

In recent decades, significant and robust large-scale changes have been observed in atmospheric circulation over Antarctica and the Southern Ocean [80–85]. These changes are of major importance in terms of impacts on the broader coupled atmosphere-ocean-ice system across Antarctica and the Southern Ocean [83, 86]. The dominant pattern of lower- and mid-tropospheric circulation variability at high southern latitudes is the Southern Annular Mode (SAM) or Antarctic Oscillation. A more positive phase of SAM is associated with anomalously low atmospheric pressure over Antarctica and higher pressure at lower latitudes [81, 87]. In recent years, more direct diagnostics of the circumpolar mid-latitude southern near-surface westerlies (hereinafter the westerly jet) have become an important part of quantifying and understanding SAM-related changes in hemispheric-scale circulation [88–90]. Strengthening and/or poleward shifting of the westerly jet are associated with a more positive SAM index and vice versa.

The depletion of stratospheric ozone and resulting strengthening of the polar vortex in the late twentieth century caused a major shift in Southern Hemisphere tropospheric climate, inducing a more positive SAM index mainly in the summer season (Fig. 1) [80, 81, 84, 85, 91]. The causal chain for this starts with the fact that ozone absorbs solar radiation, and therefore in sunlit seasons, the depletion of stratospheric ozone induces local radiative cooling of the polar stratospheres [92]. In response to cooling, the stratospheric polar vortex strengthens as changes in the vertical shear of zonal wind adjust to maintain thermal wind balance. Antarctic stratospheric ozone depletion is most pronounced in late winter/early spring since the chemical reactions responsible are linked to polar stratospheric clouds that form in the very cold conditions of the polar night [93]. Consequently, there is a strong seasonality in polar ozone-related stratospheric cooling trends, which are most pronounced in early spring. In observations, the resulting stratospheric circulation anomalies propagate downwards gradually during spring to exhibit the strongest impact in summer [80]. Identifying the precise mechanisms for this downward propagation is still a topic of debate [83, 94]. Despite uncertainty over the mechanisms, there is strong consensus that stratospheric ozone depletion has had an impact on tropospheric circulation based on evidence from both observations and modelling, and both in terms of the detection of the shift and its attribution to ozone depletion [95, 96]. This consensus has been increased in recent years due to the more widespread use of atmospheric models that extend high enough to include most of the stratosphere (so-called high top models), which more consistently reproduce the stratospheric impacts and downward propagation of circulation anomalies associated with stratospheric ozone depletion [97, 98]. However, the precise mechanisms for the transfer of signals from the stratosphere to the surface are still a subject of debate [94]. Accurate modelling and improved understanding of mechanisms are important for accurately simulating the relative contribution from other factors that can influence the SAM. In particular, increasing GHG have likely re-enforced the recent ozone-induced SAM increases [99]. The proportion of this contribution is, however, difficult to establish since multi-decadal climate variability has also been implicated as an important contributor to decadal time-scale SAM index trends [100]. For example, a key role for decadal cooling of the western tropical Pacific has been identified as contributing to observed positive austral summer and autumn SAM index trends that have occurred since the end of rapid ozone depletion in the late 1990s [101].

Observed changes in the SAM cannot be mapped simply onto changes in westerly jet strength, since changes in jet latitude and width also contribute to recent SAM trends [90]. This is consistent with recent idealised model studies showing that shifting and strengthening of the westerly jet respond differently to different drivers and across different seasons [102, 103••]. For example, a key aspect of behaviour that emerges from such atmospheric model experiments is that tropospheric heating on either side of the westerly jet acts to induce a change in jet strength, but heating collocated with the jet itself acts to shift the meridional temperature gradient maximum and the associated jet [37, 103••]. Ocean coupling does not appear necessary for simulating the jet shift. However, the patterns of warming and cooling under climate change are complex and include coupled ocean-atmosphere-ice feedbacks, the details of which are the subject of on-going research.

Looking to the future, further change is likely across a range of emission scenarios, but the character and even sign of these changes depends on a delicate balance between the relative contributions from a range of drivers. The CMIP5 models include either prescribed or simulated stratospheric ozone recovery over the twenty-first century to near pre-ozone-hole levels by 2100. Therefore, climate models exhibit a cancellation between SAM index decreases associated with stratospheric ozone recovery and SAM index increases associated with increased GHG and global warming. The most up-to-date studies indicate an approximate cancellation of effects for medium emission scenarios, mainly in summer (Fig. 1), but for high emission scenarios, a dominance of GHG driving overall SAM increases across all seasons by the year 2100 [89, 99, 104]. However, for a given scenario, the CMIP5 models still produce a wide range in projected changes [88]. Diversity across different climate models in simulations of future changes in the SAM has been linked to a range of different processes including: uncertainties in the representation of shortwave radiative forcing (associated with clouds) [105]; the representation of eddy feedbacks [106, 107]; and uncertainties associated with the response of the coupled ocean-atmosphere-ice system across different timescales [108–111]. In terms of the coupled system, recent research has shown that the jet strength component of projected westerly change is highly correlated with biases in Antarctic sea ice across the CMIP5 models [112]. Models with excessive sea ice in their baseline climate simulate more retreat in the future and more polar amplification. This stronger polar amplification weakens the lower-tropospheric temperature gradient across the storm track and acts to cancel out some of the strengthening of the westerlies that occurs due to other processes (in particular, increasing upper-tropospheric temperature gradients). This indicates a potential avenue for constraining future projections of the westerlies based on observed baseline climate, although improved understanding of the mechanisms involved is required.

### Southern Stratospheric Polar Vortex

Although the southern polar vortex is situated at high altitudes in the stratosphere, the dramatic changes that have occurred in it over recent decades have been important for many of the surface climate changes observed over Antarctica and the Southern Ocean [83]. There is a very pronounced annual cycle in the polar vortex, which is strongest in winter. The very low temperatures associated with the strong winter vortex provide conditions conducive to stratospheric ozone depletion by ozone depleting substances, as solar radiation returns to polar latitudes in early spring. Since ozone absorbs solar radiation, anomalously strong ozone depletion in spring leads to anomalously cold conditions in the mid-to-lower polar stratosphere. The formation of the ozone hole during rapid depletion between the 1970s and late 1990s is estimated to have produced a spring cooling of approximately 6 K and an associated strengthening of the polar vortex of 5–6 m s^−1^ [80, 88].

Since approximately the year 2000, Antarctic stratospheric ozone depletion appears to have ended, with signs of possible recovery [113•]. Projections to the end of the twenty-first century generally indicate recovery approaching pre ozone-hole levels, but that lower-stratospheric cooling associated with greenhouse-gas induced global warming prevents full recovery due to lower temperatures in winter acting to increase the occurrence of polar stratospheric clouds, which enhances depletion of stratospheric ozone during the return of sunlight in spring [114, 115]. In isolation, the near-recovery of stratospheric ozone concentrations would result in a near reversal of the recent changes in the polar vortex in spring. The precise rate and eventual extent of ozone recovery and related reversal in polar vortex changes differ across different climate models with implications for projections of twenty-first century surface climate change. In particular, there is significant model uncertainty over the extent to which stratospheric ozone recovery may act to cancel out projected future greenhouse gas-induced poleward jet shifts [116].

### Amundsen Sea Low

The Amundsen Sea Low (ASL) is a climatological low-pressure centre, which migrates seasonally west towards the Ross Sea in winter and east towards the Bellingshausen Sea in summer [117]. It is a key influence on variability of Antarctic-wide zonal wave numbers 1 and 3 since it forms one of the troughs of climatological wave-3 and also occurs in the vicinity of the ridge of wave-1 [118]. Regionally, changes in the depth and location of the ASL have a major impact on the climate of West Antarctica [119, 120] and it is therefore important in the broader context of understanding observed trends in regional sea ice and West Antarctic ice sheet mass balance [121].

Analyses of past variability indicate that the ASL has deepened in recent decades in association with the increasingly positive polarity of the SAM. However, the drivers of change in the ASL are particularly complex to understand since it is situated in the region of largest atmospheric pressure variability in the Southern Hemisphere [122]. Consistent with this, observations and model results show that a significant part of changes in the post-1979 modern satellite era can be linked to tropical variability. Most notably, El Niño and La Niña are associated with weakening and intensification of the ASL, respectively, in association with a Rossby wave train from the central Pacific that is most prominent in winter. This Rossby wave train is a key explanation for the occurrence of the Pacific South American Pattern (PSA), which is a winter-dominated pattern of troughs and ridges that connects ENSO variability in the tropical Pacific with West Antarctica and the Antarctic Peninsula [123] and in particular, the depth of the ASL [117, 119]. However, more recently, the importance of the Pacific Decadal Oscillation (PDO) in spring has been highlighted in association with a wave train propagating from further west in the tropical Pacific that is consistent with a deepening of the ASL linked to the recent trend towards a negative polarity of the PDO [124]. The importance of tropical variability relative to external drivers such as GHG in explaining recent trends was highlighted by Schneider and Deser (2017), who found that historically forced climate model simulations only reproduced observed ASL deepening when tropical sea surface temperature variability was constrained to observed variability [125••]. Some climate models do, however, exhibit significant deepening of the ASL in response to stratospheric ozone depletion alone [126, 127], so it is possible that both tropical drivers and stratospheric ozone have contributed significantly.

Looking at future climate change projections, Hosking et al. (2016) found that out of a subset of 11 CMIP5 models, selected based on skill at representing the ASL, the majority simulate a poleward migration of the ASL over the twenty-first century austral summer and autumn [128•]. Although less consistent, around half of the selected models also exhibit eastward shifts in autumn and winter. These changes are less consistent than the hemispheric-scale strengthening and poleward shift of the westerly jet that are simulated across the CMIP5 ensemble under the same future scenario, thus illustrating the greater uncertainty in projections of smaller-scale regional circulation change around Antarctica.

### Sub-Antarctic Cyclones

The geography of the southern polar region differs significantly from its northern counterpart. This means that, distinct from those in the Northern Hemisphere, in the Southern Hemisphere the storm tracks and regions of heightened baroclinicity lie over, or very close to, the sea ice pack [129]. In addition, the circulation is much more zonally symmetric in the high southern latitudes. These and other factors mean that sea ice and large-scale atmospheric drivers might influence the behaviour of storms in different ways from those in the Northern Hemisphere. Cyclone activity is well correlated with the SAM, with more cyclones south of 60°S and fewer north of this latitude during the positive SAM phase [58, 130, 131].

Grieger et al. (2018) undertook a comprehensive analysis of sub-Antarctic cyclones making use of 14 state-of-the-art tracking methods applied to the ERA-Interim reanalysis [132]. Robust positive trends in cyclone numbers were identified by almost all methods for austral summer over the hemispheric region south of 60°S, mainly due to the strong relation to SAM, and in the Amundsen-Bellingshausen Seas sector. Fewer methods indicated significant positive trends over the East Antarctica and Weddell sectors. Trends in austral winter were positive for most regions and methods, but only statistically significant using some tracking methods.

The Antarctic region presents an environment somewhat different to that of the Arctic for cyclone properties simulated by models for the twenty-first century. Consistent with the Northern Hemisphere, Tamarin-Brodsky and Kaspi (2017) highlighted a summer poleward shift by 4.9° latitude of maximum zonally averaged cyclone track density and a notable increase in track density in the sub-Antarctic between 55 and 65°S [78••]. Very marked in their results is a strong poleward expansion of the cyclogenesis region, with genesis increasing markedly in the 60–70°S band in response to the simulated shift in baroclinicity. With significant changes predicted in baroclinicity and total column water vapour [133] in the southern polar region, one would expect changes in the frequency of extreme cyclones. Indeed, the CMIP5 models depict significant increases in extreme cyclones (defined in numerous ways) around most of the periphery of the Antarctic continent [134].

An indirect measure of the changes in sub-Antarctic cyclone behaviour can be obtained by examining phenomena that are directly influenced by these synoptic systems. Most of the Antarctic precipitation falls on the peripheral areas of the ice sheet, and this component of the precipitation is mainly influenced by sub-Antarctic cyclones and fronts. Palerme et al. (2017) investigated the changes, between 1986–2005 and 2080–2099, in annual Antarctic precipitation simulated by the CMIP5 models [135]. They found model-average increases ranging from 5.5 to 24.5% for low to high emission scenarios, implying greater cyclonic activity and/or more precipitation per cyclone due to increased moisture availability.

### Comparing the Arctic and Antarctic

It is informative to briefly compare and contrast the two polar regions. Despite differing geography, the atmospheric circulations of the northern and southern hemispheres share a number of common features, including stratospheric polar vortices, tropospheric westerly jets and associated storm-tracks. There is broad hemispheric symmetry in how the polar circulation responds to well-mixed GHG. In both regions, increased GHG acts to shift poleward the westerly jets and storm-tracks, leading to more positive NAM and SAM indices.

A major source of hemispheric asymmetry is the role of stratospheric ozone depletion, which is much larger in the Antarctic. In recent decades, the reinforcing effects of increasing GHG and ozone depletion have led to a robust Southern Hemisphere circulation changes, especially in austral summer. Anticipated ozone recovery may also exert a strong influence, but in the opposite sense, partly or fully offsetting changes to the increasing GHG. Another key difference over recent decades has been the contrasting changes in sea ice. Whilst Arctic sea ice has declined rapidly, there has been a small overall increase in Antarctic sea ice [136]. Related to this, the Arctic has warmed at a much faster rate than the global average—a phenomenon known as Arctic amplification. Arctic amplification is understood to be important influence on winter circulation change in the Northern Hemisphere [23–26]. In contrast, the Southern Ocean has warmed slower than the global average. Although models suggest that Antarctic amplification will occur over the coming centuries, due to slow adjustment of the ocean circulation, its emergence is delayed relative to the Northern Hemisphere [137]. Accordingly, sea ice loss and polar amplification appear to be more important in modulating near-term circulation change in the Arctic than in the Antarctic.

## Conclusions

The most pronounced atmospheric circulation changes over the twentieth and twenty-first century to date have occurred in the Southern Hemisphere. The southern stratospheric polar vortex has strengthened and the SAM has shifted towards its positive phase (Fig. 1), most strongly in austral summer, associated with a poleward migration of the westerly jet. These trends are largely attributable to human influence, specifically, emission of GHG and ozone-depleting substances. The surface expression of the effects of Antarctic ozone depletion and increased GHG has reinforced one another, mainly in austral summer, driving the SAM trend over recent decades. Stratospheric ozone depletion may also have played a role in the observed deepening of the ASL; however, separating human influence from that of natural variability is harder than for the SAM. In the Northern Hemisphere, changes in the stratospheric polar vortex and NAM are within the bounds of natural variability and cannot be attributed to human-induced climate change, despite speculation that these changes are caused by Arctic sea-ice loss.

Future projections of the NAM and SAM reflect the balance of multiple and opposing climate drivers. CO_2_ concentrations are projected to continue to rise, at least until 2050 under the most-ambitious mitigation scenarios [1]. As noted earlier, there are signs that Antarctic ozone concentrations are beginning to rise; however, increasing emissions of short-lived anthropogenic chlorocarbons could slow this recovery [138]. Thus, over coming decades, ozone recovery and GHG increases will likely have opposing effects on the SAM in austral summer. For the NAM, the effects of GHG and Arctic sea ice loss appear to oppose each other, particularly in boreal winter. Thus, in both hemispheres, what happens to the annular modes will reflect the balance of competing influences. Whilst the influence of GHG on the SAM and NAM is relatively consistent across seasons, the influence of Arctic sea ice loss on the NAM is greatest in boreal winter and the influence of ozone recovery on the SAM is greatest in austral summer. Thus, NAM and SAM projections are more uncertain in boreal winter and austral summer, respectively (Fig. 1). Outside of these seasons, the effect of GHG is dominant, leading to more robust projections of increasingly positive NAM and SAM.

Past changes in Arctic cyclones vary considerably by season and by region and are sensitive to the data set, time period and methods used. Overall, there is some evidence for a change in the frequency or behaviour of Arctic storms. Furthermore, storms are occurring in a changing larger-scale environment. Moist intrusions associated with the passage of cyclones into the Arctic appear to be becoming more common, owing to increased humidity of the source air masses. Despite model uncertainty, future projections tend to support a reduction in the number of Arctic cyclones in boreal winter and more frequent storms in boreal summer, reflecting opposite trends in baroclinicity in these seasons. In the sub-Antarctic, an observed increase in cyclones south of 60°S is closely related to the positive SAM shift.

We have highlighted some of the progress being made in understanding how climate change is manifested in polar atmospheric circulation. This will remain a key area of scientific enquiry, due to substantial remaining uncertainties and because of the critical role that atmospheric circulation plays in setting both the magnitude of global climate change—e.g. SAM affects Southern Ocean CO_2_ sequestration [139, 140]—and the character of regional climate change [3].
